# A Review: Proteomics in Retinal Artery Occlusion, Retinal Vein Occlusion, Diabetic Retinopathy and Acquired Macular Disorders

**DOI:** 10.3390/ijms18050907

**Published:** 2017-04-28

**Authors:** Lasse Jørgensen Cehofski, Bent Honoré, Henrik Vorum

**Affiliations:** 1 Department of Ophthalmology, Aalborg University Hospital, Hobrovej 18-22, 9000 Aalborg, Denmark; henrik.vorum@rn.dk; 2 Department of Clinical Medicine, Aalborg University, Søndre Skovvej 15, 9000 Aalborg, Denmark; 3 Department of Biomedicine, Aarhus University, Ole Worms Allé 3, Building 1182, 024, 8000 Aarhus C, Denmark; bh@biomed.au.dk

**Keywords:** age-related macular degeneration, biological marker, diabetic retinopathy, mass spectrometry, proteomics, retina, retinal vein occlusion, vascular endothelial growth factor, vitreous body

## Abstract

Retinal artery occlusion (RAO), retinal vein occlusion (RVO), diabetic retinopathy (DR) and age-related macular degeneration (AMD) are frequent ocular diseases with potentially sight-threatening outcomes. In the present review we discuss major findings of proteomic studies of RAO, RVO, DR and AMD, including an overview of ocular proteome changes associated with anti-vascular endothelial growth factor (VEGF) treatments. Despite the severe outcomes of RAO, the proteome of the disease remains largely unstudied. There is also limited knowledge about the proteome of RVO, but proteomic studies suggest that RVO is associated with remodeling of the extracellular matrix and adhesion processes. Proteomic studies of DR have resulted in the identification of potential therapeutic targets such as carbonic anhydrase-I. Proliferative diabetic retinopathy is the most intensively studied stage of DR. Proteomic studies have established VEGF, pigment epithelium-derived factor (PEDF) and complement components as key factors associated with AMD. The aim of this review is to highlight the major milestones in proteomics in RAO, RVO, DR and AMD. Through large-scale protein analyses, proteomics is bringing new important insights into these complex pathological conditions.

## 1. Introduction

Retinal artery occlusion (RAO), retinal vein occlusion (RVO), diabetic retinopathy (DR) and age-related macular degeneration (AMD) are ocular diseases that may result in severely decreased vision or blindness [1]. The introduction of intravitreal inhibitors of vascular endothelial growth factor (VEGF) has dramatically improved the treatment of RVO, DR and neovascular AMD [2,3,4,5,6], but existing treatments require frequent injections and follow-up visits [7]. Long-term treatment of RVO, DR and AMD is often needed, as the diseases are not eliminated by existing medical treatments [6,8,9]. No effective treatment for RAO is available [1].

In order to develop treatments for RAO, RVO, DR, and AMD it is essential to achieve a better understanding of the complex pathological processes underlying these diseases. Large-scale protein studies using proteomic techniques have proved very effective in generating important knowledge about the complex pathological mechanisms underlying RVO, DR and AMD [10,11]. Furthermore, intraocular therapeutic targets have been identified with proteome studies [10].

Considering the wide use of anti-VEGF agents [12,13,14], it is interesting to observe how proteomic techniques are beginning to bring important insights into the complex protein changes that occur following anti-VEGF intervention. In the present article we review major findings of proteomic studies of RAO, RVO, DR and AMD including an overview of ocular proteome changes associated with anti-VEGF treatments.

## 2. Anatomic Overview of the Human Eye

**Figure 1:**
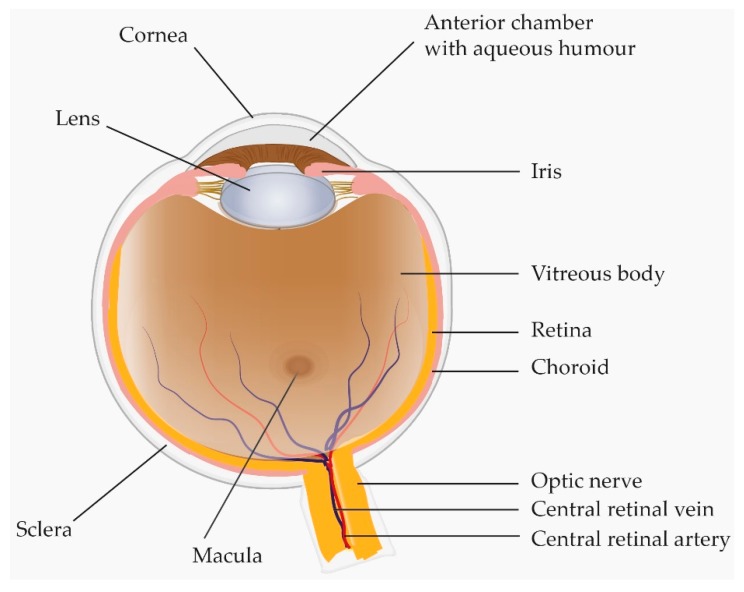
Anatomic overview of the human eye. With its close proximity to the retina and choroid the vitreous can reflect important pathological changes in chorio-retinal diseases.

The retina is a thin-layered structure that lines the innermost wall of the eye (Figure 1). The inner aspect of the retina faces the vitreous body whilst the outer aspect faces the choroid [15,16]. Due to the close anatomical and biological relationship between the vitreous body and the retina, growth factors, hormones, transport proteins and enzymes present in either structure may easily influence and be a reflection of the other [15,17,18]. At the cellular level, the retina consists of the inner multi-layered neurosensory retina, and the outer single-layered retinal pigment epithelium (RPE) [19]. The macula is a small oval-shaped area approximately 6 mm in diameter that is located between the temporal branches of the retinal vessels. A central area of the macula, the fovea, is responsible for the highest resolution of vision [19,20,21].

**Figure 2:**
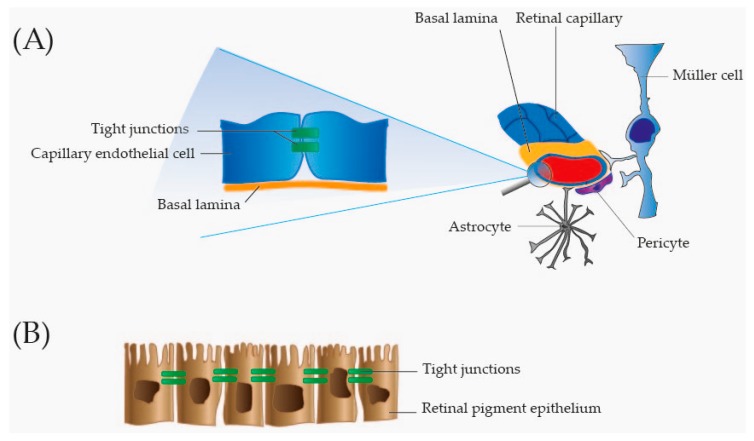
Overview of the blood–retinal barrier. (**A**) Inner blood–retinal barrier. The inner blood–retinal barrier is formed by tightly adherent endothelial cells in the retinal capillaries. Tight junctions in the intercellular spaces keep the capillary endothelial cells tightly adherent. The endothelial cells rest on a basal lamina surrounded by pericytes, and foot processes of Müller cells and astrocytes. Pericytes, Müller cells and astrocytes are involved in the maintenance of the inner blood–retinal barrier; and (**B**) Outer retinal barrier. The outer blood–retinal barrier is secured through tight junctions between retinal pigment epithelial cells.

The central retinal artery (Figure 1) supplies the inner two-thirds of the retina facing the vitreous. The inner two-thirds of the retina are drained by retinal branch veins that fuse to form the central retinal vein that leaves the eye through the optic nerve (Figure 1). The outer one-third of the retina, which does not have any vascular beds, receives its blood supply from the highly vascular choroid localized underneath the RPE [22,23,24]. An intact barrier between the retinal tissues and ocular vascular beds is essential to maintain retinal homeostasis and to protect the retina from harmful molecules in the circulation [25]. The barrier function is secured through the blood–retinal barrier that is subdivided into the inner blood–retinal barrier and the outer blood–retinal barrier. The inner blood–retinal barrier is formed by tight junctions between adjacent capillary endothelial cells (Figure 2). These endothelial cells rest on a basal lamina that is covered by pericytes and the foot processes of astrocytes and Müller cells that are involved in the maintenance and functioning of the inner blood–retinal barrier (Figure 2) [26]. The outer blood–retinal barrier is formed by tight junctions between the closely opposed RPE cells (Figure 2). In this way the RPE regulates transport between the neurosensory retina and the fenestrated capillaries of the underlying choroid [25,26]. Disruption of the blood–retinal barrier is a common phenomenon in retinal vascular diseases and neovascular AMD. Breakdown of the blood–retinal barrier may result in leakage of plasma proteins into the vitreous and retinal layers. Thus, high levels of plasma proteins may represent a compromised blood-retinal barrier rather than a biological change. The breakdown of the blood–retinal barrier may result in macular edema which is caused by accumulation of fluid in the macula [27]. Macular edema is a frequent complication in RVO, DR and neovascular AMD and is a predominant cause of vision loss [28,29].

## 3. Proteomic Workflow

**Figure 3:**
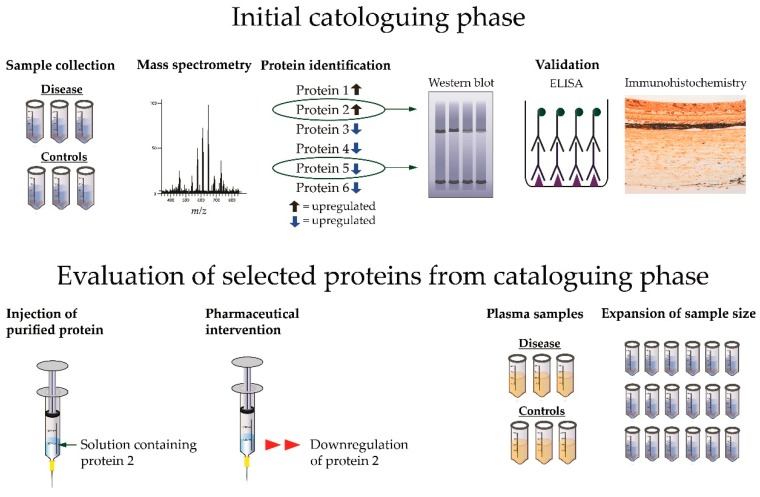
From initial cataloguing phase to evaluation of selected proteins. A typical proteomic experiment begins with collection of samples obtained from a pathological condition and appropriate controls. The samples are analyzed with mass spectrometry resulting in identification of a number proteins that are significantly changed between disease and control. Proteins of particular interest (in this example shown as protein 2 and protein 5) are often selected for validation with western blot, enzyme-linked immunosorbent assays (ELISA) or immunohistochemistry. The role in pathogenesis and the potential as a biomarker of a protein identified in the cataloguing phase can be further evaluated (in this example protein 2 is selected for further evaluation). The role of a protein in pathogenesis can be tested by injecting the protein in its purified form in an animal model or by using a pharmaceutical intervention. Another option is to test if an ocular protein change is reflected in plasma. Studies with small sample sizes may be followed-up by a new study with an expanded sample size.

The initial cataloguing phase begins with sample collection which will normally result in a set of samples from a pathological condition that is compared to relevant controls (Figure 3) [21,30]. The samples are then analyzed with mass spectrometry, allowing for identification of a large number of proteins. Proteins of particular interest which are changed in content between disease and control are often selected for validation with orthogonal analyses such as western blot and enzyme-linked immunosorbent assays (ELISA) [21] while immunohistochemistry can provide important information on the location for the observed protein changes [21] (Figure 3).

Proteins identified in the initial cataloguing phase can be evaluated to provide further insights regarding their role in the pathogenesis of a given disease and their potential as a biomarker (Figure 3). For example, the role of a protein identified in the initial cataloguing phase can be tested by using the purified form of the protein in an animal model [10]. The role of a protein identified with proteomics may be tested in animal models with pharmaceutical agents to study if the content of the protein is reversed through the intervention [31]. Proteins detected in intraocular samples from a given disease may be quantified in plasma samples to study if a given ocular disease is reflected in plasma [32]. Proteins identified in a project with a small sample size can be further evaluated by expanding the sample size [33]. For a detailed review of proteomic techniques we refer to a previous work [21].

## 4. Literature Search

The search was conducted in Ovid Medline and Ovid Embase using both controlled vocabulary terms and free text terms. The controlled terms proteome or proteomics combined with natural language terms (proteogenomic* or proteomic* or proteome*) and controlled vocabulary terms (Mesh or Emtree terms and natural language terms) for each disease were searched. The results are presented in the supplementary material. All articles using discovery proteomics were included.

## 5. Retinal Artery Occlusion

RAO can be subdivided into central retinal artery occlusion (CRAO) and branch retinal artery occlusion (BRAO). In CRAO, the central artery is occluded, while BRAO results from occlusion of a branch of the central retinal artery. Studies of the natural history of BRAO have shown that patients with BRAO have a fairly good prognosis, with 74% of patients having a visual acuity above 20/40 at onset while 89% have a visual acuity above 20/40 at follow-up [1,34]. On the other hand, CRAO is an ophthalmic emergency which often results in severe permanent vision loss, of finger counting or less. Embolism and thrombosis are the most common causes of CRAO. In the case a CRAO is caused by embolism or thrombosis, there is no effective treatment of the disease. CRAO can be a complication of giant cell arteritis which requires high doses of corticosteroids to prevent further vision loss [1,34].

**Figure 4:**
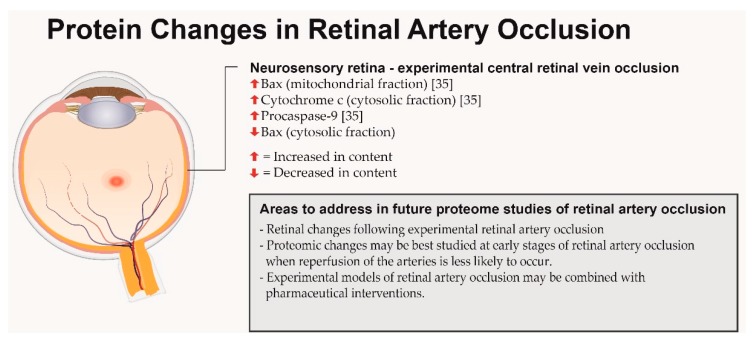
Overview of proteins associated with retinal artery occlusion. The retina is shown as a pale structure with a cherry-red spot.

To the best of our knowledge, no large-scale protein studies of CRAO have been published, but some protein changes (Figure 4) have been identified by Zhang et al. [35] who used an experimental model of laser-induced CRAO to study protein changes related to apoptosis in rat retinas. Western blot analysis was used for protein quantification. While CRAO caused decreased levels of Bax protein in the cytosolic fraction, a simultaneous increase in mitochondrial Bax protein was observed representing a shift in the Bax protein from the cytoplasm to the mitochondria. Furthermore, the contents of procaspase-9 and cytosolic cytochrome c were increased in the retina of ischemic eyes three and six hours after the occlusion had been induced.

With the poor prognosis of CRAO, proteomic studies of this disease are highly relevant. Experimental models of CRAO could be combined with proteomic techniques. In laser-induced RAO, reperfusion of the occluded arteries has been reported to occur between three to six hours after the occlusion [35]. To study retinal changes with proteomic techniques while the retinal arteries are still occluded, the retinas may be best collected at early stages of RAO.

## 6. Retinal Vein Occlusion

RVO is a major retinal disease which is subdivided into central retinal vein occlusion (CRVO) and branch retinal vein occlusion (BRVO). RVO is the second most frequent retinal vascular disease after DR. CRVO is caused by impaired outflow from the central retinal vein (Figure 1) while BRVO arises when a branch of the central vein is occluded [36]. In both CRVO and BRVO, the retina is likely to develop ischemia upstream of the occlusion, resulting in increased VEGF levels along with a multitude of inflammatory proteins that are major driving forces in the development of macular edema [37]. Visual outcome is generally moderate in both BRVO and CRVO [18,38]. The average visual acuity is generally more severely decreased in CRVO, as the entire retina is affected while a quarter to one half of the retina is affected in BRVO [1,3].

**Figure 5:**
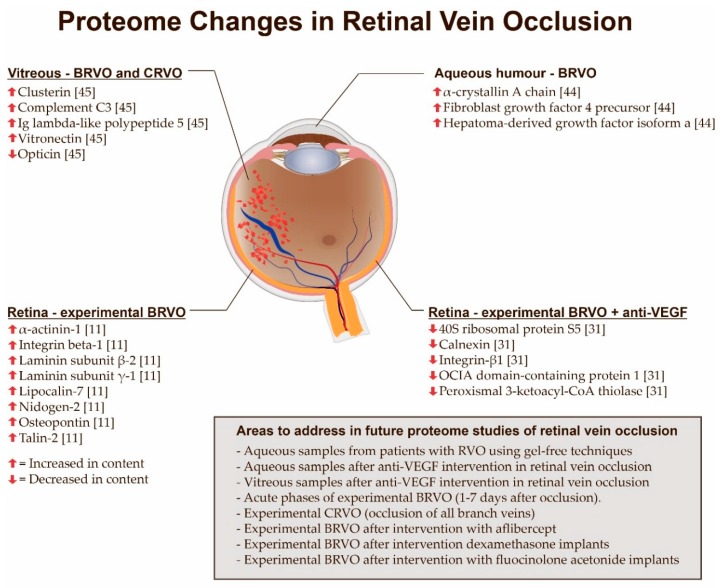
Overview of proteome changes associated with retinal vein occlusion. A branch retinal vein occlusion (BRVO) with retinal hemorrhages and venous dilation is shown. The most important cause of BRVO is arteriovenous crossing as shown in this illustration. BRVO: Branch retinal vein occlusion; CRVO: Central retinal vein occlusion; Ig: Immunoglobulin; OCIA: Ovarian cancer immunoreactive antigen; VEGF: Vascular endothelial growth factor.

Protein changes following RVO have mainly been identified with the use of enzyme-linked immunosorbent assays (ELISA) and multiplex array assays [39,40,41,42,43]. Despite a great potential to expand existing knowledge about protein changes associated with RVO, only a limited number of studies have addressed proteome changes in RVO (Figure 5). Yao et al. [44] conducted a proteomic study on aqueous humor samples obtained from patients with macular edema secondary to BRVO. These samples were compared to aqueous humor samples from patients undergoing cataract surgery. Using two-dimensional (2D) gel electrophoresis coupled with matrix-assisted laser desorption-ionization (MALDI) time-of-flight (TOF)/TOF mass spectrometry (MS) the researchers identified a downregulation of fibroblast growth factor 4 precursor, hepatoma-derived growth factor isoform a, and α-crystallin A in patients with BRVO (Figure 5). Reich et al. [45] analyzed vitreous samples from 30 treatment-naïve patients with retinal vein occlusion. Vitreous samples from RVO patients were compared to 16 controls. The samples were analyzed using a capillary electrophoresis system coupled to a micro-TOF mass spectrometer. The study found that clusterin, complement C3, immunoglobulin (Ig) lambda-like polypeptide 5 and vitronectin were significantly upregulated in vitreous samples from patients with RVO, while opticin was significantly downregulated (Figure 5). These protein changes were validated using ELISA.

Animal models of BRVO provide a unique opportunity to study proteome changes in RVO at the retinal level [11,31,46] and have revealed a number of proteins that were not previously associated with RVO. Using an experimental model of BRVO, our group [11] reported that laser-induced BRVO results in increased retinal levels of laminin subunit β-2, laminin subunit γ-1, lipocalin-7, nidogen-2, osteopontin, integrin-β1, isoform 2 of α-actinin-1, and talin-2. These findings indicated that experimental BRVO was associated with focal adhesion signaling, integrin signaling and major extracellular matrix remodeling processes (Figure 5) that are likely to take place in close proximity to the occluded vessel. The increased levels of proteins involved in integrin signaling raise the question as to whether anti-integrin therapies under development for the treatment of diabetic macular edema [47] may also be suited for the treatment of RVO. Proteome changes in experimental BRVO following intervention with anti-VEGF therapy are discussed in Section 9.

### Future Directions in Proteomic Studies of Retinal Vein Occlusion

Many areas of proteome changes in RVO remain unstudied (Figure 5). Aqueous humor samples from patients with BRVO have been studied with 2D gel electrophoresis, but a different and complimentary technique such as label-free liquid chromatography tandem mass spectrometry (LC-MS/MS) may potentially add further depth to the proteomic findings [21]. Animal models of BRVO hold a potential to bring further insights into the proteome of RVO. For example, there is very limited knowledge about retinal changes in the acute phases of experimental BRVO (approximately one to seven days after occlusion).

Future studies of experimental BRVO may address retinal changes following recently introduced anti-VEGF agents like aflibercept. Furthermore, large-scale retinal protein changes in experimental BRVO following intervention with dexamethasone intravitreal implants and long-lasting fluocinolone acetonide implants remain to be studied with proteomic techniques. While the retinal proteome in BRVO is currently under study, future studies may seek to uncover the retinal proteome of experimental CRVO, which could be induced by photocoagulation of 3–4 major branch retinal veins in a porcine model.

## 7. Diabetic Retinopathy

DR is the leading cause of blindness among working-age individuals in developed countries and the most common complication of diabetes [48]. After 20 years of disease, almost all patients with type 1 diabetes, 80% of patients with insulin treated type 2 diabetes and 50% of patients with type 2 diabetes who do not require insulin treatment will have some degree of DR [49]. Based on clinical findings DR is classified into mild, moderate and severe non-proliferative diabetic retinopathy (NPDR) and proliferative diabetic retinopathy (PDR) [50,51]. PDR is a severe sight-threatening stage which is characterized by formation of retinal neovascularisations. Vascular leakage following DR may result in diabetic macular edema which is a sight-threatening complication if left untreated [50]. Diabetic macular edema may occur at any stage of DR, but it predominantly occurs in advanced stages of the disease [49].

**Figure 6:**
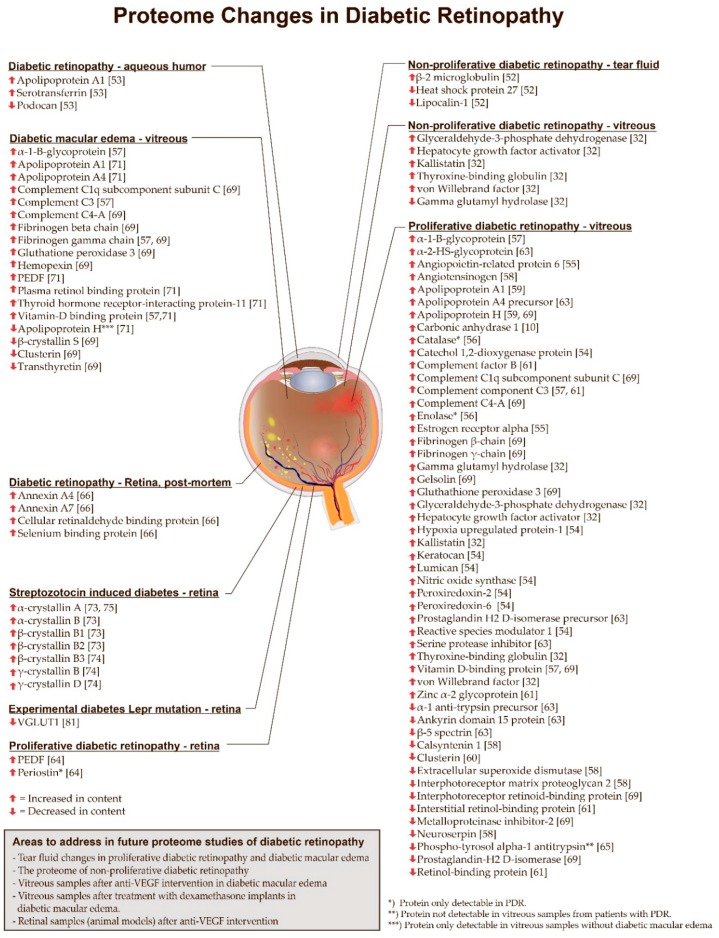
Overview of proteome changes associated with diabetic retinopathy described in the present review. The eye is presented with hemorrhages, hard exudates, cotton-wool spots and neovascularisations at the optic nerve head and in areas without relation to the optic nerve head. Furthermore, diabetic macular edema is shown in the figure. PEDF: Pigment epithelium derived factor. VGLUT: Vesicular glutamate transporter 1.

Proteomic studies of DR have led to the identification of proteome changes involved in the pathological processes that take place in the development of the disease (Figure 6). Furthermore, proteomic analysis of samples from patients with DR have succeeded in the identification of potential therapeutic targets [10].

### 7.1. Non-Proliferative Diabetic Retinopathy

The proteome of NPDR has been studied using a wide variety of materials including tear fluid, aqueous humor and vitreous body (Figure 6). Studies of tear fluid have the major advantage that tear fluid is widely available and does not require any intraocular surgery. Kim et al. [52] conducted a proteomic study on tear fluid from patients with diabetes mellitus without retinopathy, patients with NPDR and healthy volunteers. With the use of 2D gel electrophoresis coupled to the study identified an upregulation of β-2 microglobulin and a downregulation of lipocalin-1 and heat shock protein 27 in diabetic patients without retinopathy and in NPDR when compared to healthy controls. The fold changes were most pronounced in patients with NPDR.

Chiang et al. [53] found increased levels of serotransferrin and apolipoprotein A–I in aqueous humor samples from patients with DR, whilst the level of podocan was found to be decreased.

Kim et al. [32] studied vitreous samples from patients with PDR and NPDR which were compared to vitreous samples from patients with macular holes using multiple reaction monitoring (MRM). Thyroxine-binding globulin, kallistatin, hepatocyte growth factor activator, von Willebrand factor, and glyceraldehyde-3-phosphate dehydrogenase were increased in both PDR versus macular holes and NPDR versus macular holes (Figure 6). The study also found that γ-glutamyl hydrolase was found to be increased in PDR versus macular holes, but decreased in NPDR versus macular holes (Figure 6). The experiments were followed conducting an MRM analysis of plasma samples. The study found no difference between PDR and macular holes. However, thyroxine-binding globulin was increased in plasma from patients with NPDR compared to patients with macular holes. A validating western blot analysis revealed that plasma thyroxine-binding globulin was significantly increased in NPDR compared to controls and in diabetes without retinopathy.

### 7.2. Proliferative Diabetic Retinopathy

The proteome of PDR has been more intensively studied than the proteomes of NPDR and diabetic macular edema (Figure 6). The majority of proteomic studies of PDR have been conducted on vitreous samples.

The proteome study of PDR with the largest sample size was conducted by Loukovaara et al. [54] who used label-free LC-MS/MS. Vitreous samples were collected from patients with PDR and NPDR, reaching a total sample size of 138 vitreous samples. By comparing vitreous samples from patients with PDR to vitreous samples from patients with NPDR, the study revealed an upregulation of the extracellular matrix proteoglycans lumican and keratocan which had not earlier been associated with PDR. The authors hypothesized that lumican and keratocan may mediate recruitment of chemokines in the inflammatory response caused by PDR. The authors also identified an upregulation of proteins involved in oxidative stress and reactive oxygen species including peroxiredoxin-2, peroxiredoxin-6, reactive species modulator 1 and catechol 1,2-dioxygenase protein. Furthermore, hypoxia-upregulated protein-1 and nitric oxide synthase were upregulated in PDR samples.

In vitreous samples from patients with PDR, Wang et al. [55] identified an upregulation of angiopoietin-related protein 6 and estrogen receptor-α which the authors hypothesized to be involved in angiogenesis and vascular permeability associated with PDR.

In a study by Yamane et al. [56] using 2D gel electrophoresis vitreous samples from patients with PDR were compared to vitreous samples from patients with macular holes. The study found that enolase and catalase were only detectable in vitreous samples from patients with PDR.

Shitama et al. [57] compared vitreous samples from patients with PDR to vitreous samples from patients with macular holes and fibroretinal membranes. The study identified a number of proteins that were significantly increased in PDR. The fold change of fibrinogen-γ was particularly pronounced as the fold change was 173-fold. Other proteins increased in PDR included α-1-B-glycoprotein, complement component C3 and vitamin D-binding protein.

Gao et al. identified [58] angiotensinogen as an important driving force in the development of PDR. By comparing vitreous samples from patients with PDR, diabetic patients without retinopathy and non-diabetic patients, the study found that angiotensinogen was upregulated in PDR compared to samples from diabetic patients without retinopathy and non-diabetic patients. Proteins that were decreased in PDR compared to diabetic patients without retinopathy included neuroserpin, interphotoreceptor retinoid-binding protein, extracellular superoxide dismutase, interphotoreceptor matrix proteoglycan 2 and calsyntenin 1.

Proteomic studies normally do not focus on the type of diabetes underlying DR. Nevertheless, Simó et al. [59] analyzed vitreous samples from PDR due to type 1 diabetes which were compared to vitreous samples from patients with macular holes. Using 2D fluorescence difference gel electrophoresis (2D-DIGE) coupled to MALDI-TOF-MS, the researchers identified increased intravitreal levels of apolipoprotein A-I and apolipoprotein H in PDR.

A number of proteomic studies have identified changes in pigment epithelium-derived factor (PEDF) following PDR. Wang et al. [60] studied vitreous samples from patients with PDR which were compared to vitreous samples from normal eyes donated for corneal transplantation. The samples were analyzed using 2D-DIGE. The study identified significantly lower levels of PEDF and clusterin in vitreous samples from patients with PDR. This finding was validated by western blot. García-Ramirez et al. [61] used 2D-DIGE to study vitreous samples from patients with PDR using vitreous samples from patients with macular holes as controls. In vitreous samples from patients with PDR, the researchers identified increased levels of complement component 3, complement factor B and zinc α-2-glycoprotein, as well as a downregulation of interstitial retinol-binding protein and PEDF. Decreased PEDF levels could represent a shift towards angiogenesis as in RVO [62], but the role of PEDF in PDR remains poorly understood as proteomic studies of PDR have reported conflicting results. For example, Kim et al. [63] analyzed vitreous samples from patients with PDR undergoing surgery for tractional retinal detachment. With 2D gel electrophoresis followed by identification with MALDI-TOF MS and MALDI-TOF MS/MS, the study found increased vitreous levels of PEDF, serine proteinase inhibitor, prostaglandin-H2 d-isomerase precursor, apolipoprotein A-IV precursor and α-2-HS-glycoprotein. Significantly reduced proteins included α-1 anti-trypsin precursor, β-5 spectrin and ankyrin repeat domain 15 protein. Takada et al. [64] obtained PDR specimens that were compared to epiretinal membranes. The study found that the content of PEDF was significantly increased in PDR specimens. Thus, it is possible that vitreous PEDF levels are different in PDR complicated by tractional retinal detachment compared to PDR without tractional retinal detachment. The study also found that periostin was only identified in PDR specimens. A follow-up reverse transcription polymerase chain reaction (RT-PCR) analysis showed that periostin expression in PDR was significantly higher than in epiretinal membranes.

In general, there is little knowledge about changes in the phosphoproteome of DR, but one study of phosphoproteomes in vitreous samples from patients with PDR was conducted by Mukai et al. [65]. Vitreous samples from PDR patients were compared to vitreous samples from patients with retinal detachment and patients with macular holes. Proteins were separated by sodium dodecyl sulfate polyacrylamide gel electrophoresis (SDS-PAGE) and visualized using a Phos-tag kit. The study found that phosphotyrosol α-1 antitrypsin was not identified in vitreous samples from patients with PDR.

A study of the proteome in PDR by Gao et al. [10] is a great example of how proteomic techniques are able to identify useful potential targets. The study compared the proteomes of vitreous samples from patients with PDR, diabetic patients with no retinopathy, and non-diabetic patients. The study revealed a significant upregulation of vitreous carbonic anhydrase-I in vitreous samples from patients with PDR and diabetic patients with no retinopathy compared to non-diabetic patients. The results were followed up by injecting purified human carbonic anhydrase-I into the rat vitreous, which resulted in increased retinal vascular permeability. The effect of carbonic anhydrase-I on the retinal vascular permeability was widely inhibited through a co-injection of acetazolamide or methazolamide, which both inhibit carbonic anhydrase-I. Thus, proteomic techniques identified carbonic anhydrase-I as a protein that causes increased retinal vascular permeability in diabetic retinopathy. Nevertheless, the potential of inhibitors of carbonic anhydrase-I for the treatment of PDR remains to be evaluated.

### 7.3. Retinal Diabetic Changes in Eyes Obtained at Post-Mortem

Retinal samples from patients with DR are rarely obtained at post-mortem. Nevertheless, Decanini et al. [66] obtained retinal pigment epithelium of human donors with DR (stage not specified) which were compared to human retinal pigment epithelium samples from human diabetic donors with no DR. Using 2D gel electrophoresis, the study revealed a number of proteins that had not earlier been associated with DR. These included cellular retinaldehyde binding protein, annexin A4, annexin A7 and selenium binding protein, which were all increased in content.

### 7.4. Diabetic Macular Edema

Diabetic macular edema is a major cause of vision loss [67] which may require several intravitreal injections and follow-up visits to achieve successful treatment and stabilization [68]. Therefore, identification of potential pharmaceutical targets with proteomic techniques is highly relevant. Until now proteome studies of diabetic macular edema have been focused on changes in the vitreous body (Figure 6). Using 2D-DIGE, Hernández et al. [69] studied vitreous proteome changes related to diabetic macular edema. The researchers identified four vitreous proteins that were significantly different in patients with diabetic macular edema when compared to patients with PDR and macular holes. The study found that vitreous hemopexin was upregulated in diabetic macular edema compared to PDR and macular holes. β-crystallin S, clusterin and transthyretin were significantly downregulated in vitreous samples from patients with diabetic macular edema compared to PDR and macular holes. The study also identified proteins associated with PDR by comparing vitreous samples from patients with PDR to vitreous samples from patients with macular holes. Proteins that were significantly increased in PDR included apolipoprotein H, gelsolin and vitamin D-binding protein. Proteins with a lower abundance in PDR included interphotorecpetor retinoid binding protein, metalloproteinase inhibitor 2 and prostaglandin-H2 d-isomerase. The study also identified a number of proteins that were upregulated in diabetic macular edema, as well as PDR compared to controls. These proteins included complement C1q subcomponent subunit C, complement C4-A, fibrinogen β-chain, fibrinogen γ-chain and gluthatione peroxidase 3.

Shitama et al. [57] analyzed vitreous samples from patients with NPDR and diabetic macular edema which were compared to vitreous samples from patients with retinal detachment samples using with 2D gel electrophoresis coupled to MALDI-TOF-MS. Significantly increased proteins in NPDR and diabetic macular edema vs. retinal detachment included α1-B-glycoprotein, complement component C3, fibrinogen γ-chain and vitramin D-binding protein. Complement component C3 was 52-fold upregulated compared to controls. Complement activation in DR is still poorly understood [70], but proteomic studies clearly indicate that complement activation has a role in the pathogenesis of DR.

Vitreous proteome changes associated with diabetic macular edema in NPDR patients were also studied by Ouchi et al. [71]. With 2D gel electrophoresis coupled to LC-MS/MS, vitreous samples from 14 patients with NPDR and diabetic macular edema were compared to vitreous samples from four patients with NPDR and no macular edema. In diabetic macular edema, the study identified elevated levels of PEDF, apolipoprotein A1, apolipoprotein A4, thyroid hormone receptor interacting protein-11, plasma retinol-binding protein and vitamin-D binding protein. Apolipoprotein H was found to be exclusively present in samples without diabetic macular edema. The elevated PEDF level indicates that diabetic macular edema has different underlying molecular mechanisms compared to macular edema in RVO, in which PEDF levels have been found to be downregulated [72].

### 7.5. Animal Models of Experimental Diabetic Retinopathy

Animal models of diabetes allow for the study of retinal tissue exposed to experimental diabetes. In proteomic studies, streptozotocin-induced diabetes is the most frequently used experimental model [73,74,75,76,77] (Figure 6). Overall, there has been good consistency in proteomic studies of streptozotocin-induced diabetes. Three independent studies have identified increased contents of retinal crystallins following streptozotocin-induced diabetes [73,74,75]. These crystallins included α-crystallin A [73,75], α-crystallin B, β-crystallin B1, β-crystallin B2 [73] β-crystallin B3, γ-crystallin B and γ-crystallin D [74]. VanGuilder et al. [73] proposed the hypothesis that crystallins serve to protect neurons and glia of the retina from inflammatory damages.

Gao et al. [76] studied changes in the proteome of murine retinas following streptozocin-induced diabetes. Retinal proteins were separated by SDS-PAGE and analyzed with LC-MS/MS. When retinas from diabetic rats were compared to retinas from healthy rats, a total of 65 proteins were differentially changed more than 2-fold in diabetic mice. Of these proteins, 23 proteins were reversed in mice treated with candesartan. Proteins that were reversed by candesartan included proteins involved in lipid metabolism including fatty acid synthase, long-chain-fatty-acid—CoA ligase 3, estradiol 17-β-dehydrogenase 8, retinol dehydrogenase 12, NADH-cytochrome b5 reductase 3, 17-β-dehydrogenase 8, retinol dehydrogenase 12, and NADH-cytochrome b5 reductase 3. Reversed proteins were also involved in apoptosis regulation including apoptosis inhibitor 5, dynamin-like 120 kDa protein and NADH-ubiquinone oxidoreductase 75 kDa subunit. An important implication of this study using candesartan intervention is that it provides a molecular background for findings observed in large clinical trials. The angiotensin receptor blocker candesartan has been demonstrated to reduce the incidence of DR in patients with type 1 diabetes mellitus [78] while candesartan treatment has been found to reduce DR progression by 34% in patients with type 2 diabetes mellitus and mild to moderate retinopathy [79].

As laser photocoagulation is a standard treatment of PDR [80] its effect on the retinal proteome is relevant to explore. Quinn et al. [77] studied retinal proteome changes following retinal photocoagulation in Dark Agouti rats with streptozotocin-induced diabetes. In the intervention group, rats received laser photocoagulation after 4 weeks of hyperglycemia, while rats in the diabetic control group were left untreated. Using 2D-gels the study found that claudin-12 and enolase 1-α were only expressed in retinas that had been treated with laser. Laser intervention resulted in a downregulation of Wnt-5-β, calretinin and LEK-1.

Ly [81] et al. analyzed retinal membrane proteomes from diabetic mice (homozygous for Lepr^db^ mutation). One cohort of the mice was treated with metformin, while the other cohort was left untreated. The study identified vesicular glutamate transporter 1 (VGLUT1) to be significantly decreased in mice with diabetes. This finding was confirmed with transcriptomic and immunohistochemical analyses. VGLUT1 was not normalized by metformin. The role of VGLUT1 in DR remains poorly understood.

Proteomic techniques combined with animal models have proved well-suited for studying therapeutic agents that are thought to have a beneficial effect on DR. For example, the effects of phlorizin were tested in an animal model of type 2 diabetes in a study by Zhang et al. [82]. The study used mice which were homozygous for the diabetic gene (db) which encodes for a G-to-T mutation of the leptin receptor. The lack of leptin signaling causes hyperphagia and obesity resulting in hyperglycemia [83]. Diabetic db/db mice were compared to mice which were heterozygous for the db gene, which does not result in diabetes. Using isobaric tags for relative and absolute quantification (iTRAQ) coupled to LC-MS/MS. The study identified a downregulation of glutaredoxin-3 in retinas of db/db mice, which was back-regulated to normal after phlorizin therapy.

### 7.6. Future Directions in Proteomic Studies of Diabetic Retinopathy

Studies based on tear fluid samples from patients with DR have shown promising results [52]. As tear fluid samples are relatively easy to obtain, these samples may be suitable material to use in future studies. Tear fluid from patients with DR has already been studied with 2D gel electrophoresis. Therefore, a complementary technique like label-free LC-MS/MS will be suited for future studies in order to gain further depth into the proteome of DR. Proteomic differences between vitreous proteomes from patients with PDR and NPDR have been studied with label-free LC-MS/MS using very large sample sizes [54]. Therefore, future studies may use different proteomic techniques when comparing vitreous samples from patients with PDR and NPDR. As seen in Figure 6 the proteome of PDR has been studied intensively. Therefore, future studies may focus on the proteome of NPDR. Proteome changes in diabetic macular edema following intervention with anti-VEGF therapies or dexamethasone implants remain largely unelucidated and may be addressed in future studies. Furthermore, proteome changes following interventions with anti-VEGF therapies may be studied in animal models of streptozocin-induced diabetes.

## 8. Age-Related Macular Degeneration

AMD is a leading cause of blindness in the elderly throughout the world [84]. AMD can be further subdivided into dry AMD and neovascular AMD (also referred to as wet AMD). Early stages of dry AMD are characterized by the presence of drusen, which are deposits of extracellular material localized at the RPE–choroidal interface [85]. Intermediate AMD involves the presence of drusen that are confluent and larger than in early AMD. In patients with extensive drusen in the macula there is a major risk that dry AMD can progress to neovascular AMD [86,87]. Neovascular AMD represents an advanced form of AMD and accounts for approximately 10% of all cases of AMD. Neovascular AMD results from the formation of choroidal neovascularisation (CNV). A number of processes are known to be involved in the development of CNV including migration of endothelial cells of the choriocapillaris to the RPE monolayer and into the neurosensory retina. The choroidal endothelial cells proliferate and develop into CNV by a complex mechanism that involves a loss choriocapillaris that is thought to generate a hypoxic stimulus [84]. Clinical manifestations of neovascular AMD include accumulation of retinal, subretinal and sub-RPE fluid. If left untreated, neovascular AMD will typically result in severe vision loss [88,89].

### 8.1. Dry Age-Related Macular Degeneration (AMD)

**Figure 7:**
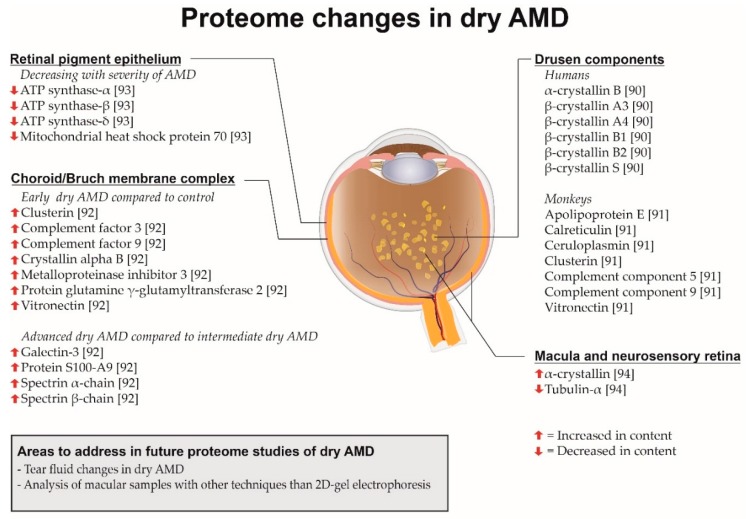
Overview of proteome changes associated with dry age-related macular degeneration (AMD) described in the present review. Drusen are seen as yellow spots.

Proteome changes in dry AMD have been analyzed studying the composition of drusen from patients with dry AMD (Figure 7). Crabb et al. [90] studied 18 normal eyes and five eyes from human donors with clinically diagnosed AMD. Drusen were isolated and analyzed with LC-MS/MS. The study found that tissue metalloproteinase inhibitor 3, clusterin, vitronectin and serum albumin appeared to be common in drusen from patients with no AMD while crystallins were more frequently identified in drusen from patients with AMD. These crystallins included α-crystallin B, β-crystallin B1, β-crystallin A3, β-crystallin A4, β-crystallin B2 and β-crystallin S. Furthermore, the study found that carboxyethyl pyrrole immunoreactivity identified by Western blot was more frequent in drusen from patients with AMD than in normal donor eyes suggesting that oxidative protein modifications have a key role in the pathogenesis of AMD. The protein composition of drusen has also been studied in animals. Umeda et al. [91] studied the proteome of drusen in late onset and early onset macular degeneration in monkeys which corresponds to early stages of dry AMD in humans. The study found that apolipoprotein E, complement component 5, complement component 9 and vitronectin were components of drusen isolated from monkeys. These findings were confirmed by immunohistochemitstry combined with autofluorescence. Other drusen constituents included the oxidative stress reactants calreticulin and ceruloplasmin. The presence of complement in drusen supports the role of complement activation as a driving force in the development of AMD.

Knowledge about the proteome of dry AMD has also been generated by studying the choroid/Bruch membrane complex in human eyes obtained at post-mortem (Figure 7) as done by Yuan et al. [92]. AMD donor eyes were from clinically diagnosed patients and obtained between 1.5 and 7 h after death while normal control eyes were obtained from an eye bank. The samples were analyzed using iTRAQ and strong cation exchange (SCX) chromatography followed by MS. The study identified eight proteins that were significantly increased in AMD compared to control donor eyes. These quantitative differences were confirmed using Western blots. The proteins included metalloproteinase inhibitor 3, vitronectin, complement factor 3, complement factor 9, clusterin, α-crystallin B and protein-glutamine γ-glutamyltransferase 2. About 60% of the elevated proteins in AMD were associated with immune response and cellular defense processes supporting the role of inflammatory processes in AMD. The study found a number of proteins that were significantly elevated in advanced dry AMD compared to early/mid stage dry AMD. These proteins included protein S100-A9, galectin-3, spectrin α-chain and spectrin β-chain.

The role of mitochondrial protein in dry AMD was studied by Nordgaard et al. [93] who obtained RPE from human donor eyes. The study found that mitochondrial heat shock protein 70, ATP synthase-α, ATP synthase-β and ATP synthase-δ decreased with increasing severity of AMD. According to the authors the decreased ATP synthase levels support the hypothesis that mitochondrial dysfunction is involved in the pathogenesis of AMD.

Ethen et al. [94] obtained samples of the neurosensory retina post-mortem from human donors with AMD. Samples were obtained from the macula as well as the peripheral retina. The study found that α-crystallin was increased with severity while tubulin decreased with severity of AMD in the macular region as well as the peripheral retina.

### 8.2. Neovascular AMD

**Figure 8:**
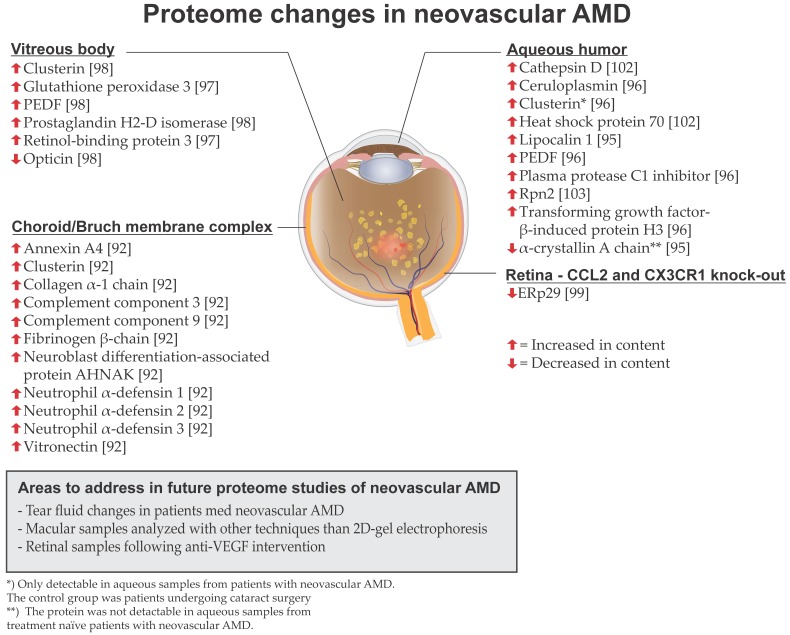
Overview of proteome changes associated with neovascular AMD described in the present review. The illustration shows an eye with drusen, macular hemorrhages and macular edema.

Proteomic studies of neovascular AMD have been conducted on aqueous humor, vitreous and the choroid/Bruch membrane complex (Figure 8). In aqueous humor samples from treatment-naïve patients with neovascular AMD, Yao et al. [95] identified a 3-fold upregulation of lipocalin-1 using 2D gel electrophoresis coupled with MALDI-TOF and TOF/TOF MS. Furthermore, α-crystallin A chain was not detectable in aqueous samples from treatment-naïve neovascular AMD patients. Kim et al. [96] analyzed aqueous humor samples from patients with neovascular AMD which were compared to control patients with no ischemic ocular disease who underwent cataract surgery using nano-electrospray ionisation (ESI)-MS/MS. The study identified significantly increased abundances of plasma protease C1 inhibitor, ceruloplasmin, PEDF and transforming growth factor-β-induced protein-H3. These selected proteins were verified using MRM.

Koss et al. [97] analyzed 73 vitreous samples from patients with treatment-naïve neovascular AMD that were compared to 15 vitreous samples from patients with idiopathic floaters. Vitreous proteins were identified using capillary electrophoresis coupled to MS as well as MS/MS. The majority of significantly upregulated proteins in neovascular AMD were plasma proteins. The study identified an upregulation of retinol-binding protein 3, which is located in the extracellular matrix between the retinal pigment epithelium and the photoreceptors where the protein binds retinoids. Glutathione peroxidase 3 was also elevated in vitreous samples from patients with neovascular AMD, which was suggested to indicate oxidative stress in neovascular AMD. Nobl et al. [98] analyzed 108 vitreous samples from patients suffering from neovascular AMD, which were compared to 24 vitreous samples from patients with idiopathic floaters using capillary electrophoresis coupled to MS. In vitreous samples from patients with neovascular AMD, the researchers identified an upregulation of clusterin, PEDF and prostaglandin H2-D isomerase, while opticin was found to be downregulated in neovascular AMD. The findings were confirmed using LC-MS/MS and validation with ELISA.

Yuan et al. [92] studied proteomic changes of the choroid/Bruch membrane complex from patients with different stages of AMD (study also described in the section on dry AMD). In the choroid/Bruch membrane complex from with patients with neovascular AMD the study found increased levels of neutrophil α-defensins 1–3, vitronectin, collagen α-1 chain, fibrinogen β chain, complement C3 and neuroblast differentiation-associated protein AHNAK. Furthermore, annexin A4 and complement C9 were more abundant in Bruch membrane/choroid complexes from patients with neovascular AMD compared to patients with dry AMD. Proteomic studies support the hypothesis that complement activation has an important role in the AMD pathogenesis. At the current stage, complement inhibitors are being tested for the treatment of AMD [70].

Tuo et al. [99] generated a murine strain with a knock-out of the *CCL2* and *CXCR1* genes. *CCL2* encodes for a chemokine and *CX3CR1* encodes for a chemokine receptor. The model results in development of AMD-like retinal alterations including retinal pigment epithelium changes, photoreceptor degeneration and in some cases choroidal neovascularization. Proteome analysis by 2D-gel electrophoresis coupled with LC-MS/MS identified a decreased expression of endoplasmic reticulum resident protein 29 (ERp29). As ERp29 is located in the endoplasmic reticulum, it has been hypothesized that decreased ERp29 levels may result in the formation of incompletely folded aggregates such as drusen [100].

### 8.3. Clusterin, Prostaglandin H2-D Isomerase and Pigment Epithelium-Derived Factor (PEDF)

PEDF, clusterin and prostaglandin H2-D isomerase have been found to be differentially regulated across a number of independent studies addressing proteome changes in neovascular AMD. Thus, there is solid evidence that these proteins are changed in content in neovascular AMD. PEDF has been found to be upregulated in aqueous humor [96] and in vitreous samples [98] from patients with neovascular AMD. In CNV, the upregulation of PEDF is thought to be driven by VEGF [98]. Clusterin has been found to be upregulated in vitreus samples [98] and in the choroid/Bruch membrane complex [92] in patients with neovascular AMD. Furthermore, proteomic analyses of aqueous humor have found that clusterin was only detectable in aqueous humor from patients with neovascular AMD and not in controls undergoing cataract surgery [96]. It has been hypothesized that increased intraocular levels of clusterin in neovascular AMD can be ascribed to neurogenerative disease [98]. Two independent proteomic studies have found prostaglandin H2-D isomerase to be upregulated in vitreous samples from patients with neovascular AMD [97,98]. Although the protein may act as an inflammatory component in neovascular AMD, its role in the disease remains largely unelucidated [98]. As different studies have identified increased levels of PEDF, clusterin and prostaglandin H2-D isomerase in neovascular AMD, the changes in these proteins are unlikely to be a result of chance.

### 8.4. Future Directions in AMD

Proteome studies of the macular region from humans have been based on 2D gel electrophoresis. Therefore, analyses with different proteomic techniques are likely to provide further insights into proteome changes in the macular region in AMD if macular tissue can be collected from human donors post-mortem. For example, techniques like label-free LC-MS/MS and tandem mass tags require only small amounts of retinal tissue [11,31] which make them well-suited for proteomic studies of the macular region. Future studies may also address retinal proteome changes following intravitreal anti-VEGF treatment from eyes collected post-mortem, but such donor eyes may be difficult to obtain. With advances in proteomic analysis of tear fluid it will be relevant to use label-free LC-MS/MS samples obtained from patients with AMD.

## 9. Application of Proteomic Techniques Following Treatment with Intravitreal Pharmaceuticals

Intravitreal anti-VEGF agents are widely used and highly effective in the treatment of macular edema secondary to RVO, DR and neovascular AMD [101]. However, a number of recent studies have reported that long-term anti-VEGF treatment of neovascular AMD is associated with the development of retinal pigment epithelial atrophy [12,13,14]. These studies indicate that anti-VEGF agents induce a number of poorly understood protein changes that result from inhibition of VEGF signaling. Proteomic techniques have a major potential in elucidating vitreal and retinal biological processes that take place following treatment with anti-VEGF agents. So far proteome changes following intravitreal anti-VEGF treatment have been conducted using the anti-VEGF agents bevacizumab and ranibizumab.

Our group recently conducted a study [31] in which experimental BRVO was induced in both eyes of five Danish Landrace pigs. The day after the occlusion was induced the right eyes of the animals were treated with ranibizumab whilst left eyes were treated with saline water. Using LC-MS/MS as well as tandem mass tag labeling we found that retinas treated with ranibizumab had decreased contents of intregrin-β1, peroxisomal 3-ketoacyl-CoA thiolase, OCIA (ovarian cancer immunoreactive antigen) domain-containing protein 1, calnexin and 40S ribosomal protein S5 (Figure 5). Thus, the quantitative changes in these proteins were identified with two fundamentally different proteomic techniques. Using tandem mass tags the study identified reduced levels of retinal dehydrogenase 1 in retinas treated with ranibizumab. As retinal dehydrogenase 1 oxidizes retinaldehyde to retinoic acid, the most potent metabolite of vitamin A, the study raises the question if anti-VEGF treatments lower the production of retinoic acid. With tandem mass tags we found increased levels of immunoglobulin γ-1 chain C region and immunoglobulin κ-chain C region in retinas treated with ranibizumab. These immunoglobulin chains were considered be components of ranibizumab. These immunoglobulin chains were not identified with label-free tandem mass spectrometry. Thus, tandem mass tag kits may hold a potential to monitor ranibizumab levels in experimental studies.

Loukovaara et al. [54] recently published a study on vitreal proteome changes following anti-VEGF treatment with bevacizumab. The study compared five vitreous samples from patients with PDR who received intravitreal injections with bevacizumab to patients with PDR who had not received intravitreal anti-VEGF treatment. In vitreous samples from patients with PDR bevacizumab was found to downregulate proteins involved in cell adhesion including contactin-2, cadherin family member 14, filamin-c, lumican and CD180. Inhibition of VEGF-A resulted in a downregulation of proteins involved in insulin signaling including insulin-like growth factor binding protein 2, insulin-like growth factor binding protein 7 and PERQ2 [54].

Kang et al. [102] isolated exosomes from aqueous humor samples from patients with neovascular AMD. Aqueous samples were collected before intravitreal anti-VEGF therapy with ranibizumab and acquired one month after the first ranibizumab injection. The study identified an upregulation of cathepsin D in patients with neosvascular AMD which was confirmed with Western blot. The authors hypothesized cathepsin D may act as resistance to oxidative stress in neovascular AMD. Furthermore, the study identified an upregulation of heat shock protein 70 which decreased in concentration following anti-VEGF treatment with ranibizumab. This finding was confirmed using LC-MRM analysis. The authors hypothesized that heat shock protein 70 is a biomarker of cell stress in neovascular AMD.

Lee et al. [103] analyzed aqueous humor samples from patients with neovascular AMD using label-free LC-MS/MS to study the ubiquitin-proteasome system in neovascular AMD. The study identified an upregulation of 26S proteasome non-ATPase regulatory subunit 1 (Rpn2) in aqueous humor from patients with neovascular AMD. The study also found that Rpn2 was decreased following anti-VEGF treatment with ranibizumab. The researchers proposed the hypothesis that Rpn2 is involved in oxidative stress in neovascular AMD that is reduced through anti-VEGF treatment with ranibizumab.

While proteomic studies are beginning to bring important insights into the processes that occur following intravitreal anti-VEGF there is an unmet need to study to proteome changes following anti-VEGF therapy with the most recently introduced anti-VEGF inhibitors like for example aflibercept. Furthermore, large-scale protein changes following treatment with dexamethasone intravitreal implants remain largely unstudied.

## 10. Limitations in Proteomics

Discovery proteomics allows for identification of large numbers of proteins [31]. For example, modern techniques are able to identify more than 3000 proteins in porcine retinal samples [31]. However, discovery proteomics does not guarantee identification of specific key proteins. This is the case for a number of growth factors including VEGF, platelet-derived growth factor and placental growth factor which are rarely detected in discovery proteomics [11,31]. However, recent studies have proved that receptors for growth factors can be identified in experiments using discovery proteomics. For example, VEGF receptor 2 and platelet-derived growth factor receptor β were recently identified with label-free LC-MS/MS [31] in porcine retinal samples. Another limitation in discovery proteomics of RVO, DR and AMD is the detection of inflammatory proteins of particular interest. Proteins such as intercellular adhesion molecule 1 (ICAM1) and monocyte chemoattractant protein 1 (MCP1) which are responsible for recruitment of inflammatory cells are known to be involved in the development of macular edema in RVO and DR [42]. ICAM1 and MCP1 are highly relevant to monitor in RVO and diabetic macular edema, but experiments using discovery proteomics generally do not identify ICAM1 and MCP1 [31].

## 11. Conclusions

With the poor prognosis of RAO, especially CRAO, it is highly relevant to study the disease with proteomic techniques, but the proteome of RAO still remains to be studied. This could be done by combining animal models of CRAO and proteomic techniques. There is only limited knowledge about the proteome of RVO, but studies on animal models indicate that experimental BRVO is associated with extracellular matrix remodeling and adhesion processes that are at least partly reversed by anti-VEGF treatment. Proteome studies of PDR have resulted in identification of important potential therapeutic targets such as carbonic anhydrase-I. Proteomic analyses have demonstrated that angiotensin receptor II antagonists can reverse retinal proteome changes caused by streptozotocin-induced diabetes. This finding is consistent with results from clinical trials. Within proteomic studies, PDR is the most intensively studied stage in DR. Therefore, future studies of DR may focus on NPDR and diabetic macular edema. A number of independent proteome studies have identified increased levels of clusterin, prostaglandin H-2 isomerase and PEDF to be associated with neovascular AMD. With reports on long-term anti-VEGF treatment resulting in retinal pigment epithelial atrophy in neovascular AMD, it is important to further explore proteome changes associated with anti-VEGF therapy. It is highly relevant to study proteome changes caused by the most recently introduced anti-VEGF agents like aflibercept. Furthermore, proteome changes following treatment with dexamethasone intravitreal implants remain to be studied.

## Supplementary Materials

Supplementary materials can be found at www.mdpi.com/1422-0067/18/5/907/s1.

## Abbreviations

CRAO	Central retinal artery occlusion
CRVO	Central retinal vein occlusion
DR	Diabetic retinopathy
ELISA	Enzyme-linked immunosorbent assay
ERp29	Endoplasmic reticulum resident protein 29
ESI	Electrospray ionization
iTRAQ	Isobaric tags for relative and absolute quantification
Ig	Immunoglobulin
LC-MS/MS	Liquid chromatography tandem mass spectrometry
MALDI-TOF	Matrix-assisted laser desorption-ionization time-of-flight
MRM	Multiple reaction monitoring
MS/MS	Tandem mass spectrometry
MS	Mass spectrometry
OCIA	Ovarian cancer immunoreactive antigen
PDR	Proliferative diabetic retinopathy
PEDF	Pigment epithelium-derived factor
RAO	Retinal artery occlusion
RPE	Retinal pigment epithelium
Rpn2	26S proteasome non-ATPase regulatory subunit 1
RT-PCR	Reverse transcription polymerase chain reaction
RVO	Retinal vein occlusion
SCX	Strong cation exchange
SDS-PAGE	Sodium dodecyl sulfate polyacrylamide gel electrophoresis
VEGF	Vascular endothelial growth factor
VGLUT1	Vesicular glutamate transporter 1
2D	Two dimensional
2D-DIGE	Two dimensional fluorescence difference gel electrophoresis
AMD	Age-related macular degeneration
BRAO	Branch retinal artery occlusion
BRVO	Branch retinal vein occlusion
CNV	Choroidal neovascularization
ICAM	Intercellular adhesion molecule 1
MCP1	Monocyte chemoattractant protein 1
NPDR	non-proliferative diabetic retinopathy
TOF	Time of flight
