# Supplementary material for: A Review: Proteomics in Retinal Artery Occlusion, Retinal Vein Occlusion, Diabetic Retinopathy and Acquired Macular Disorders
Source: Int J Mol Sci. 2017 Apr 28;18(5):907. doi: 10.3390/ijms18050907 (PMC5454820; doi:10.3390/ijms18050907)
Supplement: Supplementary file 1 [file ijms-18-00907-s001.pdf]

Supplementary material

Retinal vein occlusion

Search output from Medline

	Controlled term or natural language term	Number of identified sources
1	Proteomics/	45790
2	Proteome/	28281
3	(Proteogenomic* or proteomic* or proteome*).mp.	98781
4	or/1-3	98781
5	Retinal Vein Occlusion/	3536
6	(Retina* and (occlusion or thrombos* or obstruction*)).mp.	10597
7	5 or 6	10597
8	4 and 7	9

Search output from Embase

	Controlled term or natural language term	Number of identified sources
1	Exp proteomics/	72571
2	Proteome/	31063
3	(Proteogenomic* or proteomic* or proteome*).mp.	108023
4	Or/1-3	108023
5	Exp retinal vein occlusion/	6632
6	(Retina* and (occlusion or thrombos* or obstruction*)).mp.	16373
7	5 or 6	16377
8	4 and 7	26

Diabetic retinopathy

Search output from Medline

	Controlled term or natural language term	Number of identified sources
1	Proteomics/	45790
2	Proteome/	28281
3	(Proteogenomic* or proteomic* or proteome*).mp.	98781
4	or/1-3	98781
5	Diabetic Retinopathy/	21986
6	(Diabet* and Retinopath*).mp.	32356
7	5 or 6	32356
8	4 and 7	117

Search output from Embase

	Controlled term or natural languange term	Number of identified sources
1	Exp proteomics/	72571
2	Proteome/	31063
3	(Proteogenomic* or proteomic* or proteome*).mp.	108023
4	Or/1-3	108023
5	Exp diabetic retinopathy/	34338
6	(Diabet* and Retinopath*).mp.	44096
7	5 or 6	44096
8	4 and 7	176

Age-related macular degeneration

Search output from Medline

	Controlled term or natural languange term	Number of identified sources
1	Proteomics/	45790
2	Proteome/	28281
3	(Proteogenomic* or proteomic* or proteome*).mp.	98781
4	or/1-3	98781
5	Exp macular degeneration/	21129
6	((Macula* or retinal) adj4 (degeneration* or atroph*)).mp.	36455
7	5 or 6	41843
8	4 and 7	196

Search output from Embase

	Controlled term or natural languange term	Number of identified sources
1	Exp proteomics/	72571
2	Proteome/	31063
3	(Proteogenomic* or proteomic* or proteome*).mp.	108023
4	Or/1-3	108023
5	Age related macular degeneration/	3649
6	((macula* or retinal) adj4 (degeneration* or atroph*)).mp.	36512
7	5 or 6	36512
8	4 and 7	209

Retinal artery occlusion

Search output from Medline

	Controlled term or natural language term	Number of identified sources
1	Proteomics/	38560
2	Proteome/	24027
3	(Proteogenomic* or proteomic* or proteome*).mp.	85308
4	or/1-3	85608
5	Exp Retinal artery occlusion/	1939
6	(retina* and (occlusion or thrombos* or obstruction*)).mp.	10327
7	5 or 6	10441
8	4 and 7	8

Search output from Embase

	Controlled term or natural language term	Number of identified sources
1	Exp Proteomics/	75218
2	Proteome/	32429
3	(Proteogenomic* or proteomic* or proteome*).mp.	111992
4	or/1-3	111992
5	Exp Retinal artery occlusion/	4264
6	(retina* and (occlusion or thrombos* or obstruction*)).mp.	16724
7	5 or 6	17059
8	4 and 7	28
